# Prescriptions

**Published:** 1897-07

**Authors:** 


NITRO-GLYCERINE FOR SCIATICA.

Troussevitch has cured several obstinate cases of sciatica by giving the following drops:

℞. Solution of nitro-glycerine

(1per cent.)................½ dr.

Tincture of capsicum.....1½ dr.

Peppermint water........3 drs.

M. Sig: Mix. Five drops thrice daily in a tablespoonful of water for the first three days, then ten drops thrice daily on the subsequent days.—*Practitioner*, lvi., 221.

AN APPLICATION FOR INFLAMMATORY TOOTHACHE.

Dr. S. Wotjoff recommends this mixture for toothache, depending on inflammation of the dental pulp:

℞. Cocaine hydrochloride...1 part
Camphor...............50 parts
Chloral hydrate... ....50 parts

M. Sig: Rub enough water with the mixture to make a clear solution; rinse the mouth with it, and insert into the cavity of the tooth a bit of cotton wet with the solution, to be retained for twenty-four hours.

VAGINITIS.

℞. Alum powdered...........1 oz.
Zinc sulphate..............1 oz.
Borax......................1 oz.
Carbolic acid..............1 oz.
Water......................6 ozs.

M. Sig: A tablespoonful to a quart of lukewarm water as a vaginal injection twice daily.—*Vanderbilt Clinic.*

